# A Model of Combined Exposure to Nicotine and Tetrahydrocannabinol *via* Electronic Cigarettes in Pregnant Rats

**DOI:** 10.3389/fnins.2022.866722

**Published:** 2022-03-16

**Authors:** Kristen R. Breit, Cristina G. Rodriguez, Samirah Hussain, Karen J. Thomas, Mikayla Zeigler, Ioanna Gerasimidis, Jennifer D. Thomas

**Affiliations:** ^1^Department of Psychology, Center for Behavioral Teratology, San Diego State University, San Diego, CA, United States; ^2^Department of Psychology, West Chester University of Pennsylvania, West Chester, PA, United States; ^3^Department of Biology, West Chester University of Pennsylvania, West Chester, PA, United States

**Keywords:** prenatal, e-cigarette, nicotine, cannabis, THC, poly-drug, co-exposure, electronic cigarette

## Abstract

Nicotine and cannabis are two of the most commonly consumed licit and illicit drugs during pregnancy, often consumed together *via* e-cigarettes. Vaping is assumed to be a safer alternative than traditional routes of consumption, yet the potential consequences of prenatal e-cigarette exposure are largely unknown, particularly when these two drugs are co-consumed. In a novel co-exposure model, pregnant Sprague-Dawley rats received nicotine (36 mg/mL), tetrahydrocannabinol (THC) (100 mg/mL), the combination, or the vehicle *via* e-cigarettes daily from gestational days 5–20, mimicking the first and second human trimesters. Maternal blood samples were collected throughout pregnancy to measure drug and metabolite levels, and core body temperatures before and after exposure were also measured. Pregnant dams exposed to combined nicotine and THC had lower plasma nicotine and cotinine levels than those exposed to nicotine alone; similarly, the combined exposure group also had lower plasma THC and THC metabolite (THC-OH and THC-COOH) levels than those exposed to THC alone. Prenatal nicotine exposure gradually decreased initial core body temperatures each day, with chronic exposure, whereas exposure to THC decreased temperatures during the individual sessions. Despite these physiological effects, no changes were observed in food or water intake, weight gain, or basic litter outcomes. The use of this model can help elucidate the effects of co-exposure to THC and nicotine *via* e-cigarettes on both users and their offspring. Understanding the effects of co-use during pregnancy is critical for improving education for pregnant mothers about prenatal e-cigarette use and has important implications for public policy.

## Introduction

The potential consequences of nicotine consumption during pregnancy on both the mother and offspring have been extensively studied. Previous research has established that prenatal nicotine exposure can lead to an increased risk of sudden infant death syndrome (SIDS) (Duncan et al., 2009), low birth weight (Ernst et al., 2001; Gatzke-Kopp and Beauchaine, 2007), long-term hypertension, obesity, lung abnormalities, and many other health complications (Bruin et al., 2010). In addition, prenatal nicotine exposure can disrupt development of a number of brain regions and consequent behavioral domains (Jamshed et al., 2020). For example, prenatal nicotine exposure may increase internalizing (Cornelius and Day, 2009; Cercone et al., 2015) and externalizing behavioral problems (Kahn et al., 2003; Gatzke-Kopp and Beauchaine, 2007; Herrmann et al., 2008), hyperactivity (Ernst et al., 2001; Kahn et al., 2003) and ADHD (Mick et al., 2002; Linnet et al., 2003; Biederman, 2005; Banerjee et al., 2007; Herrmann et al., 2008), and can impair learning and memory (Cornelius et al., 2001; Longo et al., 2014), language (Fried and Watkinson, 1990; Fried et al., 1997), and cognitive performance (Fried and Watkinson, 1988; Fried and Watkinson, 1990; Fried et al., 1992; Ernst et al., 2001; Herrmann et al., 2008; Derauf et al., 2009).

Importantly, though, the delivery devices for nicotine consumption have drastically changed. The use of electronic cigarettes (e-cigarettes) has gained extensive popularity over the past several years, even among pregnant women. Recent reports indicate that 5–14% of pregnant women report using e-cigarettes during pregnancy (Cardenas et al., 2019). This is largely due to the widespread assumption that e-cigarette use is safer than traditional smoking routes. Among pregnant women with equivalent knowledge about the dangers of traditional smoking during pregnancy, 43% report believing that vaping is a safer alternative (Mark et al., 2015).

At this time, it is unclear how consumption *via* vaping could affect fetal development, especially since the e-cigarette vehicle (propylene glycol and other chemicals) contains constituents that may, by themselves, exert damaging effects (Strongin, 2019). Unfortunately, research in this area is severely limited, but the research that does exist illustrates that prenatal e-cigarette use induces fetal changes in DNA methylation, decreases birth weight, and increases birth defects (Cardenas et al., 2019). However, no research has examined the long-term effects of prenatal e-cigarette exposure, despite requests from medical professionals (Suter et al., 2015). Given that research has shown that drug consumption *via* e-cigarettes leads to higher drug and metabolite levels in the blood, including maternal and fetal blood (Young-Wolff et al., 2020), it is possible that drug consumption *via* e-cigarettes could lead to more severe consequences than previously demonstrated with traditional cigarette use.

Moreover, the design of e-cigarettes allows for multiple substances to be consumed simultaneously, which could be more detrimental than exposure to any drug alone. For example, nicotine and cannabis are more commonly consumed together than cannabis alone among pregnant women (Gray et al., 2010; Coleman-Cowger et al., 2017). On its own, the effects of prenatal cannabis exposure are still not well-understood, despite an increase in cannabis use among women of child-bearing age in the United States (Brown et al., 2017), including pregnant women (Agrawal et al., 2019), particularly during the first trimester (Volkow et al., 2019). Reports from 2014 to 2017 estimate that 4.5% of pregnant women had consumed cannabis in the past 30 days, with over 8% of pregnant women in the first trimester consuming cannabis in the past 30 days (Young-Wolff et al., 2020). These numbers have likely continued to rise, as pregnant women may purposefully consume cannabis products to combat nausea and other pregnancy symptoms, believing that gestational cannabis use is safe (Brown et al., 2017). This assumption is indeed unfounded, as more recent research has shown that cannabis use during pregnancy can actually provoke recurrent nausea and vomiting rather than combat it (Kim et al., 2018) and can interact negatively with other required medications (Ujváry and Hanuš, 2016; Brown and Winterstein, 2019).

To date, both clinical and preclinical studies examining cannabis exposure during pregnancy have yielded inconsistent results. Results are largely dependent upon the cannabinoids consumed, timing of exposure, doses, routes of administration, and a host of other factors (Schneider, 2009; Huizink, 2014). Although outcomes vary, some recorded alterations include reduced birth weight (Fried and O’connell, 1987; Day et al., 1991; Day and Richardson, 1991; Fergusson et al., 2002; Hurd et al., 2005; Huizink, 2014) and altered emotional, behavioral, and cognitive development (Huizink, 2014). Importantly, the potency of the primary psychoactive component of cannabis, tetrahydrocannabinol (THC), has risen drastically in the past few decades from 3.4% in 1993 (Mehmedic et al., 2010; Chandra et al., 2019) to the average potency of 15–20%, with (Chandra et al., 2019) with extracts and waxes reaching 60–90%, respectively (Hammond, 2021).

Moreover, similar to nicotine consumption trends, the use of e-cigarettes to consume cannabis constituents has also gained popularity. Still, we know very little about the potential effects of vaporized cannabis consumption. Thus, the results from research from previous years may not be applicable to the levels and routes of cannabis consumption that are currently used today. Several ongoing clinical studies are addressing these questions to better understand the potential consequences of prenatal cannabis consumption in current times, including the ABCD study (Paul et al., 2019); however, the results of prospective studies of consequences of current consumption patterns will not be known for years to come.

Given that nicotine and cannabis can be easily consumed simultaneously using e-cigarette tanks, it is important to examine the possible interactive effects of co-consumption of these drugs. This is especially critical given that 40% of pregnancies in the United States are unplanned (Sedgh et al., 2014), and co-consumption is highest among child-bearing age groups. For example, one study of undergraduate midwestern college students found that 77.9% of those who used e-cigarettes to consume nicotine also used e-cigarettes to consume cannabis products (Kenne et al., 2017). Importantly, when these two drugs are co-consumed *via* e-cigarettes, it may lead to additional toxicant exposure compared to use of either drug alone (Smith et al., 2019). Thus, it is vital to examine the possible interactions of nicotine and cannabis constituents on maternal physiology, so that we can explore whether combined use could be more damaging to a developing fetus.

The current study sought to develop a model of combined prenatal exposure to nicotine and THC *via* e-cigarettes using a rodent model. Studies using rodent models can help provide quick, accessible information regarding the potential consequences of prenatal nicotine and THC exposure at the levels and routes currently being consumed. Pregnant Sprague-Dawley rats were exposed to nicotine, THC, the combination, or the vehicle during a period equivalent to the human first and second trimesters. Throughout the e-cigarette exposure period, maternal body weights, food intake, and water intake were recorded. In addition, core body temperatures and blood drug and metabolite levels were collected to determine potential pharmacokinetic effects in pregnant subjects. Following birth, the length of gestation, litter characteristics, and early developmental milestones were also recorded to monitor basic litter outcomes following prenatal e-cigarette exposure.

## Materials and Methods

**FIGURE 1:**
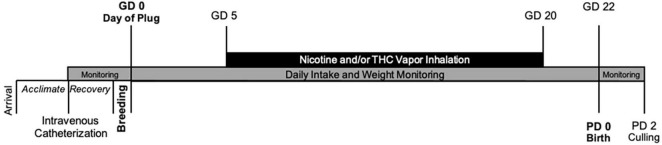
Timeline of study events.

This study developed a prenatal co-exposure model of nicotine and THC *via* e-cigarettes in pregnant rats (Figure 1). All procedures included in this study were approved by the San Diego State University (SDSU) Institutional Animal Care and Use Committee (IACUC) and are in accordance with the National Institute of Health’s *Guide for the Care and Use of Laboratory Animals*. Naïve female Sprague-Dawley rats were obtained from Charles River Laboratories (Hollister, CA) on postnatal day (PD) 60 and allowed to acclimate for at least 2 weeks prior to any handling or procedures in the animal care facilities at the Center for Behavioral Teratology (CBT) at SDSU.

### Subjects

To measure maternal blood levels of each drug and their metabolites, intravenous catheters were surgically implanted prior to pregnancy following a 2-week acclimation period, as previously published (Breit et al., 2020). Dams were anesthetized (4% isoflurane) and the catheter was secured under the skin on the back, with the tubing thread subdermally over the right shoulder and implanted into the right jugular vein *via* small incisions. Tubing was flushed (heparinized bacteriostatic saline) and cuts were sealed (VetBond; 3M). Catheters were then covered with a plastic hood and a metal screw cap to prevent chewing. Dams were administered an antibiotic (Cefazolin, 100 mg/mL; Victor Medical) and a painkiller (Flunixin, 2.5 mg/mL; Bimeda) following surgery for two consecutive days post-surgery (subcutaneous injection, 0.001 mL/g).

Following surgical recovery (∼1–2 weeks), dams were paired with a male stud to breed. Breeding pairs were housed in standard Allentown rat cages with raised grid wire floors; a filter paper was placed under the wire floor to catch any seminal plugs. Throughout breeding, pairs had *ad libitum* access to food and water. Each morning, pairs were checked for the presence of a seminal plug, which was designated as gestational day (GD) 0.

On GD 0, pregnant dams were assigned to one of four prenatal exposure groups: nicotine (36 mg/mL), THC (100 mg/mL), the combination, or the vehicle (propylene glycol). Power analyses were conducted with InStat software and indicated 10 subjects per exposure group were needed; sample sizes were determined using previously published data (Breit et al., 2020) and were conducted with alpha = 0.05 and 1-beta (power) = 0.80. Additional dams were generated to ensure power in the case of pregnancy or birth complications, or if the dam was eventually deemed to not be pregnant.

Beginning on GD 0, pregnant dams were monitored daily for gestational weight gain, as well as food and water intake. Dams were weighed prior to any procedures each morning. In addition, food and water intake from the previous day was measured and replenished each morning. Dams were given *ad libitum* access to 200 g of standard pellet lab chow (LabDiet 5001) and 400 mL of water in a graduated water bottle each day.

### Drugs

THC for e-cigarette vapor inhalation was obtained through the National Institutes of Drug Abuse Drug Supply Program and was prepared as previously described (Breit et al., 2020). Briefly, THC arrived dissolved in 95% EtOH; the THC was extracted using a SpeedVac concentrator and added to propylene glycol to obtain the chosen dose (100 mg/mL). Powdered nicotine (nicotine hydrogen tartrate salt; Sigma-Aldrich) was added to propylene glycol (Sigma-Aldrich) alone (for the nicotine group) or added to propylene glycol containing THC (combination group) and vortexed to obtain the chosen concentration (36 mg/mL). Thus, the drug solution for the combined exposure group (Nicotine + THC) contained the same amount of nicotine (36 mg/mL) and THC (100 mg/mL) as the drug solutions prepared for those that received Nicotine alone or THC alone. Doses were chosen based on current consumption patterns to mimic moderate-high plasma nicotine levels (Matta et al., 2007; Farsalinos and Polosa, 2014; Farsalinos et al., 2015; Montanari et al., 2020) and low-moderate plasma THC levels (Nguyen et al., 2016; Andrenyak et al., 2017).

### E-Cigarette Vapor Inhalation Paradigm

Vaporized drug was administered *via* commercially available e-cigarette tanks (SMOK V8 X-Baby Q2) in two 4-chamber vapor inhalation apparatuses designed by La Jolla Alcohol Research Inc. (La Jolla, CA, United States). The vapor inhalation system used a sealed cage identical in structure and dimensions as standard rat Allentown cages (Allentown, PA), a computer-controllable adapter to trigger e-cigarette tanks (SMOK V8 X-Baby Q2), and vacuum regulation of the air/vapor flow.

Vapor inhalation began on GD 5 and lasted until GD 20; this developmental period mimics the first and second trimester equivalent in humans (Enders and Schlafke, 1967). From GD 5-20, pregnant dams were placed in the vapor inhalation chambers for 40 min each day; drugs were administered through individual 6-s puffs, with puffs occurring once every 5 min through steady airflow (2 L/min) for 30 min (7 puffs total). Dams remained in the chambers for an additional 10 min to clear out any residual vapor before removal.

As demonstrated in our lab (Breit et al., 2020) and others (Nguyen et al., 2016; Javadi-Paydar et al., 2018), THC *via* e-cigarettes lowers core body temperatures in female rats. Maternal body temperatures were measured before and after each vapor exposure session *via* a rectal thermometer, to verify this expected physiological effect and to examine effects of nicotine exposure on body temperature.

Blood samples were taken *via* the intravenous catheters throughout pregnancy to determine drug levels and examine possible pharmacokinetic interactions. On several days throughout pregnancy, 700 μL of blood was taken post-vapor inhalation to establish a time curve for each drug. Prior to and after each collection, the dam’s catheter was flushed with heparinized saline (0.2 mL). In the rare case that blood could not be successfully drawn *via* the catheter, blood was instead taken *via* tail vein cut. Blood samples were immediately placed in the centrifuge to separate plasma and stored at −80°C until analyses were conducted.

Gestational lengths were recorded (usually GD 22). On postnatal day (PD) 2, the number of pups born was counted, pups’ sexes were recorded, and each pup was weighed to examine litter size, litter sex ratio, and average pup weight. Litters were pseudorandomly culled to eight pups (four females and four males, whenever possible) for future behavioral examination. Offspring were monitored daily for eye opening (both eyes fully open), a physical developmental milestone.

### Plasma Drug Analyses

THC, nicotine, and all metabolite levels were analyzed by MZ Biolabs (Tucson, AZ); samples were coded to ensure analyses were conducted by blind to treatment condition. Methodology for THC and metabolite (THC-OH, THC-COOH) analyses are described in full in Breit et al. (2020). For nicotine and metabolite (cotinine) analyses, 50 μL of plasma was precipitated using 200 μl HPLC grade acetonitrile containing 10 ng/ml nicotine-D_4_ (Cerilliant N-048-1ML) and cotinine-D_3_ (Cerilliant C-017-1ML) as internal standards. Samples were vortexed vigorously and incubated at 4°C for 30 min to precipitate proteins. After a 10-min centrifugation at 4°C, supernatant was transferred to a 96-well plate for analysis using LC-MS2. An 8-point standard curve containing nicotine (Cerilliant N-008-1ML), and cotinine (Cerilliant C-016-1ML) was prepared with concentrations of both analytes ranging from 781 pg/mL to 100 ng/mL. A Thermo Scientific Surveyor HPLC connected to a Thermo Scientific LTQ Velos Pro mass spectrometer was used for separation and quantification of nicotine and cotinine. The Surveyor sample compartment was kept at 6°C and column temperature maintained at 25°C. Analytes were separated using a 4.6 mm × 150 mm HILIC column (Phenomenex 00F-4449-E0) with isocratic flow of 500 μL/min. Isocratic conditions were 5% A, 95% B for 7 min, where A is water containing 25 mM formic acid, 1 mM trifluoroacetic acid and B is acetonitrile. Eluate was analyzed by the LTQ Velos Pro using positive ions defined in the following table. Quantitation was performed using the Quan Browser software from Thermo Scientific. An in-house QC was prepared with 10 ng/mL each of nicotine and cotinine prepared in drug free canine plasma. The linear quantitative range of the assay was from 781 pg/mL to 100 ng/mL.

### Statistical Analyses

All data were analyzed using the Statistical Packages for Social Sciences (SPSS, version 26; IBM). Data were analyzed using 2 (Nicotine) × 2 (THC) Analyses of Variance (ANOVAs) with significance levels set at *p* < 0.05. Data analyzed over multiple Days and/or Time points used these within-subject variables as Repeated Measures. One-way ANOVAs with all four groups were conducted if there were significant interactions between nicotine and THC and/or to verify specific group differences. Student Newman Keuls *post hoc* tests (*p* < 0.05) were used when needed. Offspring data additionally used Sex as a between-subject variable. For eye opening, non-parametric analyses were used. Means (M) and standard errors of the mean (SEM) are displayed visually in all graphs.

## Results

A total of 48 female Sprague-Dawley rats completed the vapor inhalation exposure procedure and gave birth (Nicotine + THC: 12, Nicotine: 12, THC: 11, Vehicle: 13). No complications were observed among pregnant dams; five dams were dropped from the study as they were not pregnant (Nicotine + THC: 0, Nicotine: 2, THC: 1, Vehicle: 2).

### Maternal Weight Gain

**FIGURE 2:**
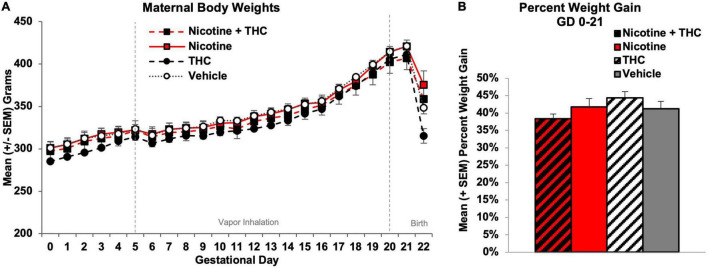
Neither maternal body weights **(A)** nor pregnancy weight gain **(B)** during gestation were significantly altered by prenatal exposure to nicotine, THC, or the combination *via* e-cigarettes.

Body weight gain among pregnant dams was analyzed using a 2 (Nicotine) × 2 (THC) ANOVA with Day as a repeated measure. Although all subjects gained weight during gestation (Day: *F*[21,924] = 1077.00, *p* < 0.001), a 2-way Day*Nicotine interaction was observed (*F*[21,924] = 1.70, *p* < 0.05). However, there were no main or interactive effects of Nicotine or THC on any individual Day (Figure 2A). Similarly, the percentage of weight gain among dams (GD 0-21) was not significantly altered by prenatal nicotine, THC, or combined exposure during gestation (Figure 2B).

To examine whether prenatal nicotine or THC exposure altered nutritional factors, food and water intake were recorded each morning (for the previous day) prior to vapor exposure. Food and water intake were analyzed separately (1) prior to the vapor inhalation exposure onset (GD 0-4) and (2) during the vapor inhalation period (GD 5-20). All intake data were collapsed over 4-day epochs for simplicity of presentation (GD 0-4, GD 5-8, GD 9-12, GD 13-16, GD 17-20).

**FIGURE 3:**
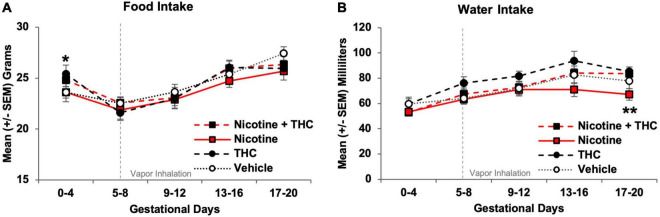
At baseline, pregnant dams assigned to receive THC exposure had higher food intakes, however, no differences among groups were observed throughout the vapor inhalation period **(A)**. During the last days of vapor inhalation, dams exposed to nicotine alone during pregnancy drank less water than dams exposed to THC, although no groups differed significantly from controls **(B)**. *THC > no THC, *p* < 0.05. **Nicotine < Nicotine + THC and THC, *p*’s < 0.05.

Prior to vapor inhalation, dams assigned to receive THC exposure (alone or in combination with nicotine) ate more food at baseline (*F*[1,44] = 4.11, *p* < 0.05), an artifact of random assignment since they had not yet received any drug exposure. Pregnant dams increased food intake throughout the vapor inhalation period (Day: *F*[3,132] = 50.45, *p* < 0.001), but neither prenatal nicotine nor THC exposure significantly altered food intake overall, across Days, or on individual Days (Figure 3A). Thus, prenatal exposures did not affect food intake.

At baseline (GD 0-4), no significant differences were seen among groups for water intake. Dams increased water intake throughout the vapor inhalation period (Day: *F*[3,132] = 23.17, *p* < 0.001); a 3-way interaction of Day*Nicotine*THC approached significance (*F*[3,132] = 2.38, *p* = 0.07) and there was a main effect of THC (*F*[1,44] = 4.63, *p* < 0.05). There were no effects of prenatal drug exposure from GD 5-16; however, by GD 17-20, pregnant dams exposed to Nicotine alone drank less water than those exposed to THC or the combination of Nicotine + THC (*F*[3,44] = 3.19, *p* < 0.05; SNK *p*’s < 0.05). However, water intake did not differ between any prenatal drug-exposed group and Vehicle controls (Figure 3B).

### Maternal Body Temperature

Maternal core body temperatures were taken before and after vapor exposure each day. Temperature data were collapsed every 4 days for presentation simplicity (GD 5-8, GD 9-12, GD 13-16, GD 17-20), and analyses used 2 (Nicotine) × 2 (THC) ANOVAs with Day as a repeated measure.

**FIGURE 4:**
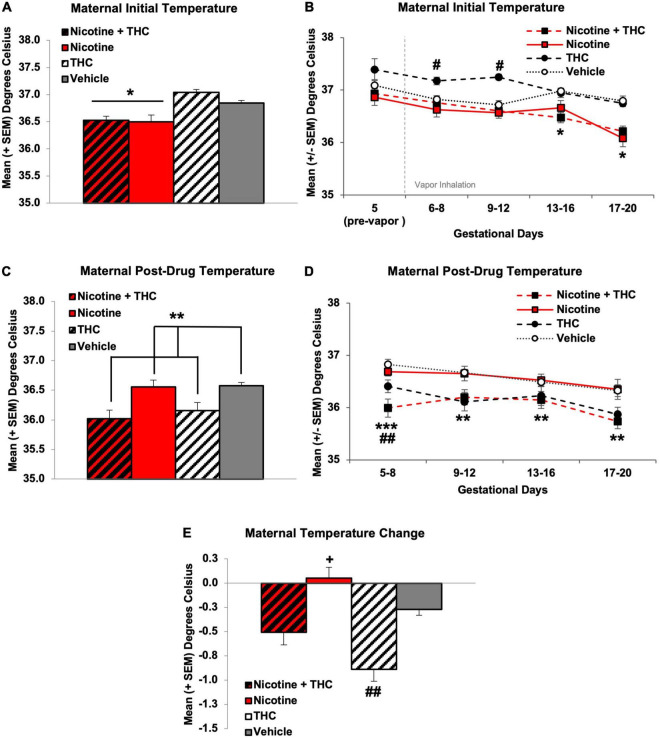
Pregnant dams exposed to nicotine *via* e-cigarettes had lower initial daily temperatures across the vapor inhalation exposure period **(A)**. At the beginning of drug exposure, dams exposed to THC had significantly higher initial daily temperatures, which declined to control levels by mid-pregnancy. Dams exposed to any nicotine maintained lower initial daily temperatures throughout the latter half of pregnancy **(B)**. In contrast, pregnant dams exposed to THC *via* e-cigarettes had lower temperatures following vapor inhalation **(C)**. Early in exposure, this effect was driven by the combined exposure group, but a main effect of THC remained throughout the rest of the exposure period **(D)**. When compared against initial daily body temperatures, pregnant dams exposed to THC had a greater temperature change compared to all other groups **(E)**. *Nicotine different than no Nicotine, *p*’s < 0.05. ^#^THC only > all other groups, *p* < 0.01. **THC < no THC, *p*’s < 0.05. ***Nicotine + THC < all other groups, *p*’s < 0.001. +Nicotine only > Vehicle, *p* < 0.05. ^##^THC only < Vehicle, *p* < 0.05.

Overall, gestational nicotine exposure *via* e-cigarettes decreased initial daily core body temperatures (*F*[1,44] = 29.06, *p* < 0.001; Figure 4A). However, core temperatures changed across Days and varied by prenatal exposure group, producing interactions of Days*Nicotine*THC (*F*[3,132] = 5.10, *p* < 0.01), Days*Nicotine (*F*[3,132] = 4.59, *p* < 0.01), and Days*THC (*F*[3,132] = 8.34, *p* < 0.001). On GD 5, prior to any vapor exposure, there were no significant differences in initial daily body temperatures. During the first half of treatment (GD 6-12), dams exposed to THC alone exhibited higher initial daily temperatures than all other groups (GD 5-8: *F*[3,44] = 5.33, *p* < 0.01, SNK *p*’s < 0.05; GD 9-12: *F*[3,44] = 12.57, *p* < 0.001, SNK *p*’s < 0.05). However, body temperatures of pregnant dams exposed to any of the drugs (combined Nicotine + THC: *F*[1,11] = 62.35, *p* < 0.001; Nicotine alone: *F*[1,11] = 37.36, *p* < 0.001; THC alone: *F*[1,10] = 66.39, *p* < 0.001) gradually decreased over the course of treatment, whereas maternal temperatures of Vehicle controls did not change throughout treatment. By the second half of the exposure period (GD 13-20), dams exposed to Nicotine *via* e-cigarette, alone or in combination with THC, exhibited significantly lower initial daily temperatures than those exposed to THC only or the Vehicle (GD 13-16: *F*[1,44] = 16.62, *p* < 0.001; GD 17-20: *F*[1,44] = 31.60, *p* < 0.001; Figure 4B).

Following intoxication, immediately after drug exposure, dams exposed to THC had lower body temperatures, as expected (*F*[1,44] = 17.19, *p* < 0.001; Figure 4C). In addition, a Day*Nicotine interaction was also observed (*F*[3,132] = 2.71, *p* < 0.05). During GD 5-8, dams exposed to combined Nicotine + THC had lower post-drug exposure temperatures than all other groups, whereas those exposed to THC alone had lower body temperatures compared to Vehicle controls, and those exposed to Nicotine alone did not differ from any groups (*F*[3,44] = 9.36, *p* < 0.001; SNK *p*’s < 0.05). However, throughout the rest of the exposure period (GD 9-20), a main effect of THC remained, as dams exposed to any THC had lower temperatures than those not exposed to THC (GD 9-12: *F*[1,44] = 14.22, *p* < 0.001; GD 13-16: *F*[1,44] = 5.17, *p* < 0.05; GD 17-20: *F*[1,44] = 13.10, *p* < 0.01; Figure 4D).

Because of the nicotine-related reductions in initial daily body temperature, when examining the temperature change (post-inhalation—pre-inhalation body temperature), dams exposed to THC alone had significantly greater temperature changes and dams exposed to Nicotine alone had significantly smaller temperature changes compared to the Vehicle controls. Dams exposed to combined Nicotine + THC had an intermediate effect, not differing significantly from controls (*F*[3,44] = 12.83, *p* < 0.001, SNK *p*’s < 0.05; Figure 4E).

### Nicotine Level Analyses

**FIGURE 5:**
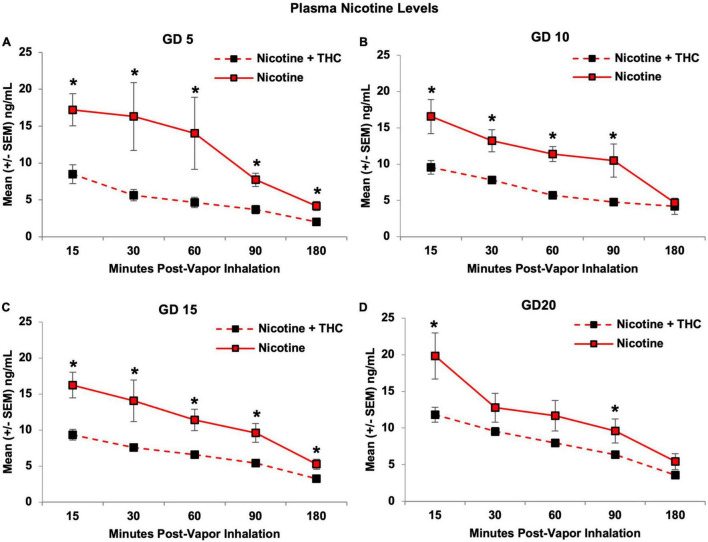
The addition of THC significantly reduced plasma nicotine levels. This effect was seen consistently on GD 5 **(A)**, 10 **(B)**, and 15 **(C)**. By GD 20, this effect was still present, but not as pronounced, as nicotine levels in the combined exposure group gradually increased throughout the vapor inhalation exposure period **(D)**. *Nicotine + THC < Nicotine, *p* < 0.05.

Dams exposed to the combination of Nicotine + THC *via* e-cigarettes had lower plasma nicotine levels than those exposed to Nicotine alone (*F*[1,19] = 37.38, *p* < 0.001; Figure 5). Group differences were most robust close to peak intoxication periods, producing an interaction of Time*THC (*F*[4,76] = 6.55, *p* < 0.01). Although the Day*Time*THC interaction did not reach statistical significance, plasma nicotine levels of dams exposed to combined Nicotine + THC, but not nicotine alone, gradually increased throughout pregnancy (*F*[3,33] = 4.99, *p* < 0.01). In fact, by the last day of vapor inhalation (GD 20), nicotine levels of dams exposed to combined Nicotine + THC were more consistent with those exposed to Nicotine alone, differing significantly only at 15 and 90 min (15-min: *F*[1,19] = 7.45, *p* < 0.05; 90-min: *F*[1,19] = 4.66, *p* < 0.05; Figure 5D).

**FIGURE 6:**
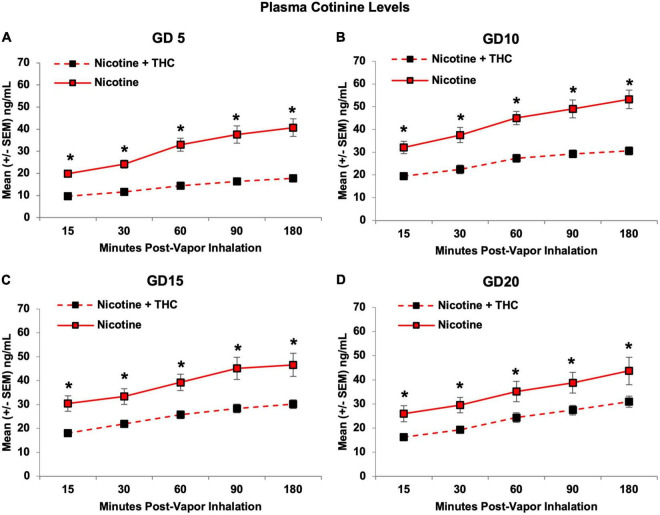
Pregnant dams exposed to the combination of Nicotine + THC had lower plasma cotinine levels than those exposed to Nicotine alone on each day and time throughout the vapor inhalation procedure **(A–D)**. *Nicotine + THC < Nicotine, *p* < 0.05.

Consistent with nicotine levels, plasma cotinine levels were also lower in pregnant dams exposed to combined Nicotine + THC compared to those exposed to Nicotine alone (*F*[1,19] = 40.20, *p* < 0.001), an effect that became less pronounced over days, producing a 3-way interaction of Day*Time*THC (*F*[12,228] = 3.53, *p* < 0.001). Although both exposure groups showed an expected rise in cotinine levels over Time (*p*’s < 0.001), only dams exposed to combined Nicotine + THC showed an increase in average levels across Days (*F*[3,33] = 4.99, *p* < 0.01). Importantly, the Nicotine + THC-exposed dams exhibited lower cotinine levels on each Day and Time point (*p*’s < 0.05; Figure 6).

### Tetrahydrocannabinol Level Analyses

**FIGURE 7:**
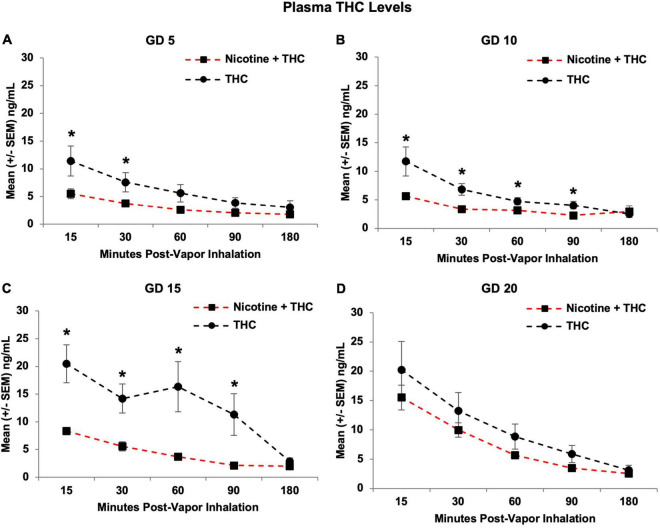
Pregnant dams exposed to the combination of Nicotine + THC had lower plasma THC levels than those exposed to THC alone on gestational days 5 **(A)**, 10 **(B)**, and 15 **(C)**. However, this effect was not seen by the last day of vapor inhalation **(D)**, as plasma THC levels gradually rose across days in both groups. *Nicotine + THC < Nicotine, *p* < 0.05.

Similarly, combined nicotine exposure reduced plasma THC levels in the pregnant dams compared to those exposed to THC alone (*F*[1,21] = 17.28, *p* < 0.001). Although THC levels increased over days in both groups, consistent with our previous research (Breit et al., 2020), this increase was more drastic in the combined group, producing a Day*Nicotine interaction (*F*[3,63] = 3.19, *p* < 0.05), as well as a Time*Nicotine interaction (*F*[4,84] = 4.46, *p* < 0.01; Figure 7). With this gradual increase, although the combination exposure group had lower overall plasma THC levels on GD 5 (*F*[1,21] = 4.20, *p* = 0.05), 10 (*F*[1,21] = 8.19, *p* < 0.01), and 15 (*F*[1,21] = 20.94, *p* < 0.001), levels caught up and did not differ significantly by GD 20.

On the first day of vapor inhalation (GD 5), dams exposed to Nicotine + THC had lower plasma THC levels compared to those exposed to THC alone at 15 (*F*[1,21] = 4.61, *p* < 0.05) and 30 min post-vapor inhalation (*F*[1,21] = 4.73, *p* < 0.05), but not past 60 min post-inhalation (Figure 7A). On GD 10 (Figure 7B) and 15 (Figure 7C), the combined exposure group had lower THC levels at all Time points (*p*’s < 0.05) except for 180 min post-vapor inhalation. However, by GD 20, the exposure groups no longer differed at any Time point (Figure 7D).

**FIGURE 8:**
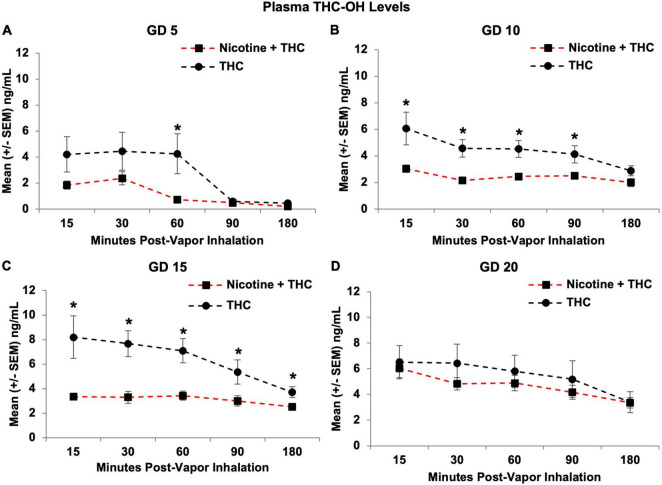
Pregnant dams exposed to the combination of Nicotine + THC had lower THC-OH metabolite levels than those exposed to THC alone on gestational days 5 **(A)**, 10 **(B)**, and 15 **(C)**. However, this effect was not seen by the last day of vapor inhalation **(D)**, as metabolite levels gradually rose in the combination group. *Nicotine + THC < Nicotine, *p* < 0.05.

Consistent with THC levels, pregnant dams exposed to the combination of Nicotine + THC also had lower THC-OH metabolite levels than those exposed to THC alone (*F*[1,21] = 7.87, *p* < 0.05; Figure 8), except on GD 20, producing a Day*Nicotine interaction (*F*[3,252] = 3.19, *p* < 0.05). Over the Days, THC-OH levels gradually increased among both dams exposed to Nicotine + THC (Day: *F*[3,33] = 24.43, *p* < 0.001) and THC alone (Day: *F*[3,30] = 5.17, *p* < 0.05). The combination exposure group had lower metabolite levels on GD 5 (*F*[1,21] = 4.27, *p* = 0.05), 10 (*F*[1,21] = 9.14, *p* < 0.01), and 15 (*F*[1,21] = 13.48, *p* < 0.01), but caught up to THC only group levels on GD 20. In addition, a Time*Nicotine interaction (*F*[4,84] = 4.88, *p* < 0.01) confirmed that the combined exposure group had lower THC-OH levels at earlier time points (15, 30, 60, and 90 min post-vapor inhalation; *p*’s < 0.05), but not toward the end of the sessions.

Follow-up analyses confirmed that on the first day of vapor inhalation (GD 5), dams exposed to Nicotine + THC had lower THC-OH levels compared to those exposed to THC alone only at 60 min post-vapor inhalation (*F*[1,21] = 5.69, *p* < 0.05; Figure 8A). On GD 10, the combined exposure group had lower metabolite levels at all Time points (*p*’s < 0.05) except for 180 min post-vapor inhalation (Figure 8B); but by GD 15, this difference was observed at all Time points (*p*’s < 0.05; Figure 8C). Yet, by GD 20, the exposure groups no longer differed at any Time point (Figure 8D).

**FIGURE 9:**
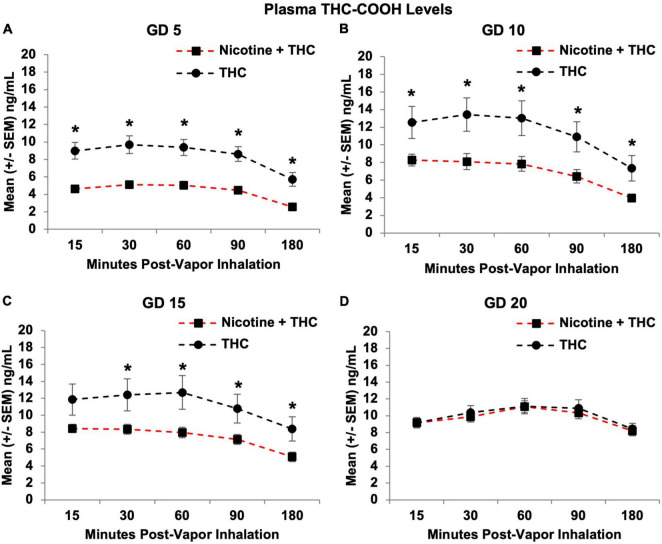
Pregnant dams exposed to the combination of Nicotine + THC had lower THC-COOH metabolite levels than those exposed to THC alone on gestational days 5 **(A)**, 10 **(B)**, and 15 **(C)**. However, this effect was not seen by the last day of vapor inhalation **(D)**, as metabolite levels gradually rose in both exposure groups. *Nicotine + THC < Nicotine, *p* < 0.05.

Similar effects were observed in the THC metabolite levels for THC-COOH; THC-COOH levels were lower in pregnant dams exposed to the combination of Nicotine + THC compared to those exposed to THC alone (*F*[1,21] = 13.82, *p* < 0.01), but this varied by Day (Day*Nicotine: *F*[3,63] = 2.69 *p* < 0.05) and Time point (Time*Nicotine: *F*[4,84] = 2.45, *p* = 0.05; Figure 9). Over the Days, plasma THC-COOH levels gradually increased among dams exposed to Nicotine + THC, but not among those exposed to THC alone. Thus, the combination exposure group had lower overall THC-COOH levels on GD 5 (*F*[1,21] = 21.52, *p* < 0.001), 10 (*F*[1,21] = 8.05, *p* < 0.015), and 15 (*F*[1,21] = 4.86, *p* < 0.05), but not on GD 20.

Follow-up analyses indicated that dams exposed to Nicotine + THC had lower THC-COOH levels at each Time point on GD 5 (*p*’s < 0.01; Figure 9A) and GD 10 (*p*’s < 0.05; Figure 9B). On GD 15, the combined exposure group had lower THC-COOH levels at all Time points (*p*’s < 0.05) except for the earliest Time (15; *p*’s < 0.05; Figure 9C). By the last day of vapor inhalation (GD 20), the exposure groups no longer differed at any Time point (Figure 9D).

### Litter Outcomes

Despite the effects on core body temperature and blood levels, prenatal exposure to nicotine, THC, or the combination did not alter basic litter outcomes. No significant differences were observed among gestational lengths, the number of pups born, the sex ratio of litters (Female:Male), or average pup weights at birth (Table 1). Similarly, no differences were observed in the developmental milestone of eye opening (Table 1).

**TABLE 1:** Neither prenatal exposure to nicotine nor THC altered basic litter outcomes or the first day of full eye opening.

Prenatal exposure group	Gestation length (days)	Litter size (number of pups)	Sex ratio (females/males)	Pup weight (grams)	Eye opening (day)
	(M ± SEM)	(M ± SEM)	(M ± SEM)	(M ± SEM)	(M ± SEM)
Nicotine + THC	21.92 ± 0.08	11.58 ± 0.56	1.11 ± 0.22	8.41 ± 0.21	14.46 ± 0.16
Nicotine	22.17 ± 0.11	12.08 ± 0.58	1.63 ± 0.45	7.94 ± 0.19	14.33 ± 0.14
THC	22.00 ± 0.00	13.09 ± 0.61	2.09 ± 0.70	8.10 ± 0.14	13.95 ± 0.14
Vehicle	21.92 ± 0.08	12.00 ± 0.61	1.24 ± 0.41	8.36 ± 0.23	14.23 ± 0.17

## Discussion

The current study sought to establish a co-exposure model of nicotine and THC *via* e-cigarette vapor inhalation that can be used safely in pregnant rats. We exposed pregnant subjects to nicotine and/or THC *via* e-cigarettes daily from GD 5-20, mimicking the human first and second trimester states when drug consumption is most prevalent (Volkow et al., 2019). We successfully demonstrated that this model induces clinically relevant blood drug levels among pregnant rats while avoiding nutritional confounds and poor litter outcomes. To our knowledge, this is the first model to mimic clinically relevant polydrug consumption of nicotine and THC using e-cigarettes among pregnant women in a rodent model, the first to thoroughly monitor multiple maternal and fetal outcomes throughout the exposure period, and the first to demonstrate pharmacokinetic interactions of nicotine and THC in blood levels of pregnant subjects.

In our model, we did not find any changes in maternal body weight growth, daily food intake, or daily water intake resulting from prenatal nicotine or THC exposure throughout the vapor inhalation period. The daily growth and intake of pregnant women consuming either of these drugs is not typically measured, although both prenatal nicotine and THC exposure have been linked to low birth weights (Ernst et al., 2001; Gatzke-Kopp and Beauchaine, 2007; Huizink, 2014). We have previously shown that prenatal THC exposure *via* e-cigarettes at the same dose used in this study (100 mg/mL) did not alter maternal weight gain, food intake, or water intake (Breit et al., 2020); the other limited studies examining prenatal THC exposure have not reported these measures. Previous research has shown that chronic exposure to cigarette smoke (Wager-Srdar et al., 1984) or nicotine alone (Grunberg et al., 1986) in non-pregnant rats reduces body weight, as well as food and water intake, but these outcomes have not yet been measured in pregnant rats, or when e-cigarette exposure is used. No consistent or significant behavioral changes were observed among dams following vapor sessions. Importantly, we did not observe any dams licking the sides of the cages or their own fur during the paradigm, nor did we see/feel any condensate on their fur upon removal or during the entirety of blood collection timelines. In sum, these data suggest that offspring outcome effects produced at these doses, such as behavioral alterations (Hussain et al., 2021; Rodriguez et al., 2021), are not related to nutritional confounds, nor are pair-fed controls required (Abel and Dintcheff, 1978).

Several studies have previously established that THC exposure *via* e-cigarettes decreases core body temperatures in non-pregnant (Javadi-Paydar et al., 2018) and pregnant female rats (Breit et al., 2020), similar to the results found in the current study; this confirms that the expected physiological effects of THC at this dose and *via* this route were achieved in these dams. Interestingly, body temperatures post-drug exposure were similar between the group exposed to THC only and the group exposed to both THC and nicotine, despite differences in plasma THC levels. In contrast, prenatal nicotine exposure reduced initial daily core body temperatures. When looking at the average temperature change across days, these individual effects of nicotine and THC led to an intermediate effect in the combined exposure group; it is important to clarify that the individual drug effects did not cancel each other out, but rather were a summation of the separate effects of nicotine and THC exposure *via* e-cigarette vapor inhalation.

It is unknown whether either of these phenomena are observed in pregnant women, although nicotine exposure is known to decrease skin conductance and temperature in humans (Rowe et al., 1980; Benowitz et al., 1982; Waeber et al., 1984; Bounameaux et al., 1988). In non-pregnant rodents, nicotine has been shown to decrease core body temperatures following intoxication *via* injections (Rezvani and Levin, 2004) and e-cigarette vapor inhalation (Javadi-Paydar et al., 2019). Similar changes have also been observed in primates (Taffe, 2012). Moreover, lasting effects of THC can increase body temperatures the following day (Taffe, 2012), which was also observed among our dams exposed to THC alone at the beginning of pregnancy, albeit initial daily temperatures tended to be higher in this exposure group prior to any THC exposure. In contrast, we did not find decreased temperatures following nicotine intoxication in the current study, but rather found decreased initial daily temperatures only following chronic exposure. Notably, pregnant rodents typically show a natural decrease in body temperature as pregnancy progresses (Melanie et al., 1988), although we did not see that among our control subjects. In any case, it is possible that maternal changes in body temperature could influence fetal development.

Interestingly, unlike rodent studies that have shown tolerance to THC-induced temperature changes with chronic exposure (Taylor and Fennessy, 1978; Uran et al., 1980; Tai et al., 2015; Nguyen et al., 2018, 2020a,b), there was no tolerance to THC’s temperature changes using this e-cigarette vapor inhalation paradigm. The lack of tolerance in the current study could be due to features of the administration route, chosen dose, or an interaction of the two. On the other hand, the lack of tolerance to temperature changes might suggest that this administration route is less invasive than many injection models used in preclinical studies, and may better mimic current human use.

In addition to the physiological effects on temperature, the blood levels of nicotine, THC, and their metabolites suggest that the doses of nicotine (36 mg/mL) and THC (100 mg/mL) used in our model are clinically relevant. We chose these doses based on public consumption patterns; the peak levels of nicotine in our maternal blood samples at this dose replicate those seen in humans and animal models *via* various administrative routes, including e-cigarettes (Matta et al., 2007; Farsalinos and Polosa, 2014; Farsalinos et al., 2015; Montanari et al., 2020). The peak levels of blood THC reached in this study are representative of those from low-moderate THC level products, given that high level THC products lead to blood THC levels around 100–150 ng/mL in humans (Andrenyak et al., 2017) and rats (Nguyen et al., 2016). It is, however, important to note that our peak levels were observed 15 min post-vapor inhalation, following a 10-min clearance time before subjects were removed from the chambers. Thus, blood levels were more representative of 25–30 min-post drug exposure, and we may have missed the true peak of blood levels of both drugs.

Nevertheless, the plasma THC and metabolite levels reached among dams exposed to THC alone in the current study and our previous work examining co-exposure to THC and alcohol are remarkably consistent (Breit et al., 2020), emphasizing the reliability of this co-exposure vapor inhalation model even when various drugs are utilized. Moreover, the variability of drug and metabolite levels found in this study for both nicotine and THC are similar to those previously shown among non-pregnant rats using similar e-cigarette vapor inhalation equipment (Nguyen et al., 2016; Javadi-Paydar et al., 2019; Smith et al., 2020). Lastly, THC and its metabolites also accumulated in maternal blood over days, which is consistent with clinical literature (Sharma et al., 2012) and has been shown previously in both non-pregnant (Fleischman et al., 1975) and pregnant rats (Breit et al., 2020), further reinforcing this model’s clinical relevance and consistency.

The most striking finding was the interaction of nicotine and THC on blood drug and metabolite levels. Pregnant dams exposed to the combination of nicotine and THC had lower plasma levels of nicotine, THC, and their metabolites compared to those exposed to only nicotine or only THC *via* e-cigarettes; this effect mostly subsided by the end of the pregnancy (GD 20), since drug and metabolite levels in the dams exposed to the combination approached the levels in those exposed to only one drug, following chronic exposure. Although the research is limited, it is important to note that the effects of vaporized THC during polydrug exposure appear to be drug-specific; while in the current study THC decreased plasma nicotine and metabolite levels, our previous work examining THC co-exposure with alcohol found the opposite effect, where THC actually increased blood alcohol concentrations (Breit et al., 2020). Since this is the first study to co-expose nicotine and THC *via* e-cigarettes to pregnant rats, and preclinical studies examining prenatal drug exposure rarely report maternal drug blood levels, there is no previous research to compare these findings too.

However, there is limited clinical research on co-consumption in non-pregnant individuals that may be applicable. For example, one study illustrated that users who smoked “blunts” (cannabis rolled in tobacco leaves) had significantly lower plasma THC levels compared to those who smoked “joints” (cannabis wrapped in cigarette paper) at equivalent concentrations, particularly among female subjects (Cooper and Haney, 2009). In this study, both “blunt” and “joint” use yielded similar increases in heart rate and carbon monoxide levels. It was hypothesized that this difference was due to the construction of the “blunts” vs. the “joints” since subjects may not be able to reach desired intoxication as quickly with blunts (Cooper and Haney, 2009); however, in the current study, both drugs were administered in the same e-cigarette tank simultaneously, eliminating this possibility. In contrast, other clinical research has shown that transdermal nicotine patch use while consuming cannabis cigarettes enhanced heart rate and reported levels of intoxication more than cannabis alone (Penetar et al., 2005). Similarly, nicotine has been shown to exacerbate several behavioral effects of THC in rodents (Valjent et al., 2002; Balerio et al., 2006). Co-administration of nicotine and THC has been shown to enhance c-Fos expression in various brain regions of rodents (Valjent et al., 2002), and nicotine also potentiates the discriminative effects of THC in rodents in conditioned place preference paradigms (Solinas et al., 2007).

We do know that both nicotine and THC induce the cytochrome P450 (CYP1A2) enzyme, as well as other CYP enzymes, with an additive induction effect following co-use. Thus, other drugs that are metabolized by this enzyme may have faster systemic clearance following such enzyme induction. Separately, both nicotine and THC use in combination with other substances have been shown to decrease plasma concentrations of these drugs in humans [see review by Anderson and Chan (2016)]. However, it important to note that plasma levels of drugs and metabolites during the 30 min of drug exposure are not known and could present a different profile. It is also possible that the co-administration of drugs within one e-cigarette tank may influence competition at the lungs for uptake of each drug, each drug’s bioavailability, or the absorption rate. While possible, this would not explain the consistency of body temperature changes caused by each drug, or the reduction of plasma level differences over time, with or without co-exposure. Moreover, we have found that the administrative parameters used in this study induce unique, additive, and interactive effects in the behavior of the offspring (Hussain et al., 2021; Rodriguez et al., 2021); it would be difficult to explain how the combination could produce more severe behavioral alterations in the offspring if less drug was absorbed. It is also possible that the combination of nicotine and THC may speed up drug distribution from the blood into other body tissues. The exact origin of the interaction observed in the current study is unclear and will require further investigation. It is even more intriguing, given that co-exposure effects on plasma levels of each drug were less robust or no longer present by GD 20, suggesting adaptations with chronic exposure during pregnancy. Regardless, co-exposure to nicotine and THC *via* e-cigarettes altered plasma levels of each drug.

Despite the physiological and pharmacokinetic effects observed, we found no alterations in basic litter outcomes. There were no pregnancy or birth complications following prenatal nicotine or THC exposure. Similarly, we found no alterations in gestational length, litter size, average offspring weight, the sex ratio of the litter, or the early developmental milestone of eye opening. Although this is consistent with our previous work looking at prenatal alcohol and THC exposure *via* e-cigarettes (Breit et al., 2020), it is inconsistent with some clinical research. Prenatal nicotine exposure has been linked to an increased risk of miscarriage, sudden infant death syndrome (Duncan et al., 2009), low birth weight (Ernst et al., 2001; Gatzke-Kopp and Beauchaine, 2007), and other early-life health complications (Bruin et al., 2010); these deficits have also been found following prenatal e-cigarette exposure (Cardenas et al., 2019). Although much less understood, prenatal cannabis exposure has been associated with low birth weight (Huizink, 2014). However, there are many methodological differences between these studies, including (but not limited to) dose levels, routes of administration, and timing of exposure. Nonetheless, these results suggest that this co-exposure model of nicotine and THC exposure *via* e-cigarettes in pregnant rats can be used to induce physiological changes associated with nicotine and THC consumption while avoiding critical nutritional confounds and alterations in basic litter outcomes.

There are several limitations of this study to address. First, these results only apply to the single doses of nicotine and THC used, which represent moderate-high levels of nicotine consumption and low-moderate levels of THC consumption commonly used today. Similarly, these results may not generalize to other cannabis products. We chose to initially examine effects of the primary psychoactive constituent, THC. However, cannabis contains more than 500 chemical compounds, including over 100 naturally occurring cannabinoids, such as cannabidiol (Radwan et al., 2017). Lastly, we acknowledge that the control group in this study was exposed to propylene glycol *via* e-cigarettes. We did compare data from the vehicle-exposed dams to a small pilot of non-handled, non-exposed dams and found no differences in body weight, food or water intake, gestation length, or any other litter outcome variables. Thus, we used the more appropriate controls for this study, but acknowledge that future research should consider that exposure to e-cigarette vehicles could affect fetal development (Strongin, 2019).

In sum, we have established a novel co-exposure model of nicotine and THC *via* e-cigarette vapor inhalation for use in pregnant rats. This model induced physiologically relevant effects of nicotine and THC exposure and produced clinically relevant pharmacokinetic interactions in maternal blood drug and metabolite levels. Importantly, these effects were achieved while avoiding effects on nutritional intake, maternal growth, gestational length, and litter outcomes related to maternal and fetal health. With this model, we are currently examining how combined exposure to nicotine and THC *via* e-cigarettes during gestation alters brain and behavioral development among offspring. These data will provide desperately needed information on the risks associated with e-cigarette use among pregnant women.

## Data Availability Statement

The raw data supporting the conclusions of this article will be made available by the authors, without undue reservation.

## Ethics Statement

The animal study was reviewed and approved by Institutional Animal Care and Use Committee at San Diego State University.

## Author Contributions

KRB holds first authorship based on roles in study design, project organization, data collection, data analyses, and writing of the manuscript. CGR contributed to project organization, data collection, and data analyses. SH and KJT equally contributed to data collection and analyses. MZ and IG equally contributed to analyses and writing of the manuscript. JDT holds last authorship based on roles in study design, funding acquisition, supervision, data analyses, and writing of the manuscript. All authors contributed significantly to the preparation of this submission.

## Conflict of Interest

The authors declare that the research was conducted in the absence of any commercial or financial relationships that could be construed as a potential conflict of interest.

## Publisher’s Note

All claims expressed in this article are solely those of the authors and do not necessarily represent those of their affiliated organizations, or those of the publisher, the editors and the reviewers. Any product that may be evaluated in this article, or claim that may be made by its manufacturer, is not guaranteed or endorsed by the publisher.
